# Curbing boredom in online teaching: Effects of an autonomy-oriented intervention with EAP students

**DOI:** 10.3389/fpsyg.2022.1060424

**Published:** 2022-11-10

**Authors:** Abbas Ali Rezaee, Haniye Seyri

**Affiliations:** Faculty of Foreign Languages and Literatures, University of Tehran, Tehran, Iran

**Keywords:** boredom, EAP students, autonomy-oriented intervention, online EAP classes, pedagogical intervention

## Abstract

Despite the growing body of research on boredom and its causes in face-to-face classes, little is known about how pedagogical interventions can mitigate this negative emotion. The purpose of this study was to examine boredom experienced by EAP students in online classes and investigate the effects of an autonomy-oriented intervention program on students’ boredom. The boredom scale was administered to 84 students before and after the autonomy-oriented intervention. By designing and implementing autonomy-oriented intervention based on the autonomy enhancement model, positive results were obtained with reduced levels of boredom. The results revealed that the intervention was effective and boredom was reduced to a noticeable extent. In addition, the qualitative results contributed to our understanding of the learners’ experiences throughout the intervention. We conclude the study with implications for EAP instructors to employ different pedagogical interventions to mitigate negative emotions in online EAP classes.

## Introduction

Learning English involves interaction, which in turn makes it an essentially emotional venture (Richards, 2020). It has been reported in various studies that among various emotions involved, language learners are susceptible to frequently experiencing boredom in university contexts (e.g., Sharp et al., 2017; Derakhshan et al., 2021). The Control-value Theory of achievement emotions (Pekrun, 2006; Pekrun et al., 2010) posits that learners may find themselves “in or out of control of achievement activities” (Pekrun et al., 2010, p. 534), and boredom is aroused when achievement emotions are conceptualized as not having value and as lacking control over the learning process (Acee et al., 2010; Pekrun et al., 2010).

Bored students show “lower perceived control” (Pekrun et al., 2010, p. 533). Moreover, Sharp et al. (2016) stress that less engagement and less opportunity to exert control on classroom events are among the main reasons for boredom. In the same vein, Zawodniak et al. (2021) remark that students are bored in teacher-centered classes in which students are provided with less freedom of choice. Given that, if students are provided with freedom of choice and autonomy over content and method of studying, they experience less boredom (Acee et al., 2010; Sharp et al., 2016, 2017). By promoting autonomy in learners, negative emotions like boredom would decrease and evidence from earlier research corroborates this assumption (e.g., Sharp et al., 2016; Yazdanmehr et al., 2021; Zawodniak et al., 2021).

On the other hand, with the significance of social distancing announced by World Health Organization (World Health Organization [WHO], 2020) as a result of the spread of COVID-19, online education has gained momentum (Ribeiro, 2020). As a result, higher education practices have been influenced by the changes in their delivery method, the incorporated material, mode of teaching, and also interactions (Adedoyin and Soykan, 2020). Review of literature on online classes indicates that students often feel bored in their online English language courses (Derakhshan et al., 2021; Pawlak et al., 2021). Due to the lack of simultaneous oral and visual presentation of materials (De Beni and Moè, 2003), the encoding of the input might be cumbersome in online classes and students’ concentration might waver and they might get bored during the class. Moreover, students in EAP (English for academic purposes) classes are susceptible to experiencing boredom due to teacher control and repetitive and boring activities (Zawodniak et al., 2017), disengagement (Pawlak et al., 2020a), which leads to severe negative consequences in the classroom. This aversive emotion is also tied to lower academic achievement (Mousavian Rad et al., 2022) and negative cognitive perceptions such as perceptions of time passing slowly (Goetz et al., 2014), cognitive passivism (Li et al., 2022).

Investigating the underlying factors contributing to boredom and when and how students succumb to this negative emotion would not be enough and different pedagogical interventions are required in order to reduce it to a noticeable extent (Pawlak et al., 2020a,^2021^). Due to the dire consequences of these negative emotions, an intervention program based on autonomy-oriented instruction, which reduces boredom by increasing students control over the learning process (Sharp et al., 2016; Zawodniak et al., 2021), is designed and implemented to alleviate students’ boredom in EAP classes. Moreover, paucity of appropriate pedagogical interventions is considered as a prominent factor leading to academic failure (Pawlak et al., 2021). The present study’s educational significance is justified through the autonomy-oriented intervention program as it sheds light on how teachers can deal with negative emotions and raise their sensitivity to students’ emotions in EAP classes. To best of our knowledge, few, if any, studies have taken measure to reduce boredom through a pedagogical intervention program. Therefore, the present study aims at implementing an autonomy-oriented intervention program which intends to increase the autonomy of EAP students and in turn decrease boredom experienced during online EAP courses.

## Review of the related literature

### Boredom

Learners’ emotions, positive or negative, exert influence on the process of language learning and their attitude toward what they learn (Resnik and Dewaele, 2020; Richards, 2020). Among different types of emotions, boredom is the most frequently experienced one in different educational contexts (Goetz et al., 2014). Despite being the subject of many studies in psychology, this concept has recently gained momentum in L2 (second language) research (Chapman, 2013; Kruk and Zawodniak, 2017). Boredom has been also trivialized in schools or higher education settings since teachers attribute boredom to other factors such as students’ laziness, anxiety, or personality characteristics (Macklem, 2015).

Boredom is considered as “complex, multifaceted, and evasive,” however, it can be discussed in relation to its “symptoms, causes, stability, degree of intensity, and valence and arousal” (Derakhshan et al., 2021, p. 2). This negative emotion is mainly manifested in disengagement, dissatisfaction, and negative emotional perceptions emanating from non-arousing settings (Lewinski, 2015). Bored students often feel detached from their goals and are less involved in the activities and as a result, they reflect signs of disengagement from the educational settings to which they belong (Henry and Thorsen, 2018; Zawodniak et al., 2021). As for the causes of boredom, different factors have been mentioned, among which unchallenging tasks (Larson and Richards, 1991), teacher control (Hill and Perkins, 1985), trivializing the importance of activity at hand (Pekrun et al., 2010), and having no purpose for learning (Yeager and Walton, 2011) have received attention. According to the control-value theory of achievement emotions (Pekrun, 2006; Pekrun et al., 2010), learners succumb to boredom when they attach little value to the task and when they lack control over the learning process. As for the deleterious effects of boredom, depression (Macklem, 2015), disengagement and lack of interaction (Tvedt et al., 2019), reticence (Shea, 2017), L2 speaking anxiety (Galante, 2018), demotivation (Mercer and Dörnyei, 2020), among others, have attracted empirical attention recently.

Until recently, boredom was mainly investigated indirectly and in relation to other negative emotions. For example, Kormos and Csizér (2014) probed the influence of motivational factors and self-regulatory strategies on Hungarian EFL high school students, university students, and adult learners’ autonomy. The results suggested that the efforts put by learners were pertinent to their potentials for overcoming boredom. Being the first study to explore boredom explicitly and directly in L2 classes, Chapman (2013) explored German L2 students’ beliefs about boredom and their three teachers. The results indicated that feelings about their teachers were the main predictor of boredom and also textbook activities, less challenging classes, and unengaged peers resulted in students’ boredom.

Other research projects focusing on boredom in L2 classes were conducted mainly in the Polish educational context (Kruk and Zawodniak, 2018, 2020; Pawlak et al., 2020a; Zawodniak et al., 2021), and very recently in Asian contexts (Li and Dewaele, 2020; Derakhshan et al., 2021; Li, 2021; Li et al., 2021; Nakamura et al., 2021), some of which are briefly outlined here. In their quantitative study, Pawlak et al. (2020b) investigated differences in the boredom level experienced by second- and third-year English major students. The findings suggested that second-year students experienced more boredom than third-year students due to the content provided during the 3-year program. Moreover, they indicated that factors underlying boredom were mainly uninteresting topics of discussion, negative past experiences, negative perceptions, and paucity of creativity and involvement. Regarding studies conducted in Asian context, Nakamura et al. (2021), using surveys and focus group interviews, investigated the predictors of students’ boredom in English oral communication courses. The results showed nine factors among which mismatch between internal leaner factors and external classroom factors such as activity mismatch, challenging tasks, and lack of adequate proficiency in L2 skills played influential role in students’ boredom.

### Boredom in online classes

On the other side, the sudden shift to online education due to unexpected events such as COVID-19 outbreak demands the investigation of students’ feelings such as boredom in online English classes (Pawlak et al., 2021). Three recently published articles have investigated boredom during online courses (Li and Dewaele, 2020; Derakhshan et al., 2021; Pawlak et al., 2021). Li and Dewaele (2020) collected information from 348 university students to explore the predictive role of boring online language classes. The results indicated moderate but higher levels of boredom in online classes in comparison to face-to-face classes. The analysis revealed that learners who had higher emotional intelligence and learning achievement perceptions experienced lower levels of boredom in online classes. Adopting a qualitative measure, Pawlak et al. (2021) examined the difference between online and face-to-face classes and also content-based and skill-based courses in relation to boredom. The results indicated that online classes were more boredom-inducing than face-to-face classes and found content-based classes more boring. In another recent study in Iran, Derakhshan et al. (2021) examined the causes and also remedies to boredom of English major students during online classes due to the pandemic by drawing on data from open-ended questionnaires and semi-structured interviews. It was revealed that teacher-learner related factors, computer-related factors, task-related factors, and student-related factors were the main causes of boredom. Moreover, five categories for coping with boredom including making the class livelier, improving IT infrastructure and individuals’ IT know-how, involving students in discussions and encouraging participation, improving interpersonal relationships, and changing the lifestyle were identified.

### Learner autonomy

Learner autonomy (LA) is considered to be the definitive goal of language learning process over the years (Teng, 2019) since it is inexorably tied to promoted L2 achievement (Little, 2007). According to Holec (1981), LA is one’s ability to shoulder the responsibility for their learning process which may include decision making in terms of the objectives, content, method, evaluation of the possible outcomes of learning. LA is conceptualized as “a precondition for effective learning” (Benson, 2013, p. 1), which implies that when autonomy is developed and enhanced, the process of learning languages is facilitated since the learner has taken charge of their learning.

Language teachers have the responsibility to enhance autonomy in their learners to ensure that learning takes place (Benson, 2011). It is believed that enhancement of autonomy leads to efficiently dealing with negative emotions such as boredom (Kormos and Csizér, 2014; Zawodniak et al., 2021). That said, teachers who make attempts to foster autonomy can provide opportunities which increase students’ engagement and involvement in the classroom (Reeve and Jang, 2006) which in turn will lead to lower levels of boredom (Mercer and Dörnyei, 2020). For example, Kormos and Csizér (2014) explored the effect of motivational factors and self-regulatory strategies on EFL learners’, university students’, and adult learners’ autonomous learning. The results showed that their efforts were linked to their ability to overcome boredom. They inferred that controlling negative emotions results in autonomy enhancement of learners.

To date, studies have focused on the level of boredom and also the relationship of the concept with other variables such as achievement, anxiety, etc. However, what remains underexplored is investigating the effects of pedagogical interventions on this emotion. Different researchers have called for implementing pedagogical interventions to mitigate boredom (e.g., Sharp et al., 2016; Pawlak et al., 2020a,^2021^).

### Aims and hypotheses

The present study aims at reducing the rate of boredom experienced by Iranian EAP learners through implementing an online autonomy-oriented intervention program in which learners’ autonomy will be fostered through autonomy enhancement approaches. The enhancement approaches were adopted from Benson’s (2011) model which will be explained in the “Materials and methods” section. The present study seeks to answer the following research question:

Does an autonomy-oriented intervention program reduce learners’ boredom in Iranian EAP classes?

Accordingly, this study intends to test the following hypotheses:

Autonomy-oriented intervention program has no significant effect on learners’ boredom in Iranian EAP classes. Regarding the qualitative section of the study, it is expected to gain useful information regarding the processes and strategies learners have experienced during the intervention sessions.

## Materials and methods

### Research design

The present study adopted a mixed-method design including questionnaires and reflective journals in order to measure the rate of boredom. The intervention was applied in order to mitigate boredom in EAP students. In doing so, an autonomy-oriented intervention was employed during which four different approaches to autonomy enhancement including resource-based approaches, learner-based approaches, classroom-based approaches, and curriculum-based approaches, developed by Benson (2011), were adopted to foster the autonomy of learners.

The intervention included 10 sessions with each session lasting about 90 mins and focusing on one particular approach to fostering autonomy. Each approach was implemented in two separate sessions. The first and last sessions of the intervention were allocated to completing the questionnaires to ensure that learners take enough care in responding to each question. Moreover, during the first session, the researcher illuminated the process of data collection and intervention, and briefed learners on their responsibilities during the intervention. Moreover, to tap into the inner processes through which learners developed their autonomy and reduced their boredom, they were asked to deliver weekly reflective journals to the researcher via Telegram messaging application.

### Participants and context

The present study was conducted at a university in Iran. The students partaking in this study have to take a three-credit semester-long online course which meets 4 h per week within different faculties such as faculty of Computer Engineering, Chemistry, Physics, etc. Students are normally put into these classes regardless of their majors. Since this is an intervention program and the changes need to be scrutinized meticulously, two classes with the total number of 84 students were selected.

The participants were aged between 18 and 22 years. The majority of the participants had studied English language in private institutes or at least, to improve their English language, they had read English materials apart from what they had been taught during their education at school, like reading books, newspapers or watching English programs.

Due to the pandemic, the classes were held online at the time of data collection. The online classes in Iran are held using different online platforms such as Adobe Connect, Big Blue Botton, and Skyroom among which Adobe connect was used for the purpose of this study because of the affordances it provides. This application is user friendly, but the most notable reason for which it was used is related to the breaking out mode through which the main room can be broken into different rooms in which students can do different pair-work and group-work activities.

### Instruments

The questionnaire used in the present study is a modified version of a similar instrument employed in the study conducted by Kruk and Zawodniak (2017) inclining 28 five-point Likert-scale items. However, the item-total correlation for four items was under the minimum criteria of 0.30 and they were removed from the main data. The present instrument consists of 23 seven-point Likert-scale statements, which tap into the intensity of boredom in the L2 classroom. Higher scores on each statement of the scale and also entire scale will indicate higher levels of boredom (Pawlak et al., 2020a). Moreover, the results of explanatory factor analysis for 23 items revealed factor loadings above 0.30 indicating that all items had large contributions to their underlying constructs. To ensure the content validity, two experts in Applied Linguistics were requested to check each item carefully. Despite the high proficiency level of students, all items of the inventory were translated into Persian, the learners’ mother tongue, in order to diminish the possibility of any misunderstanding. Then, the internal consistency reliability of the inventory was calculated for the learners partaking in this study and it was 0.91 based on Cronbach’s alpha. Some sample items include: “Time always seems to be passing slowly in my language classes,” “I often have to do repetitive or monotonous things in my language classes,” “It seems that English classes are the same all the time; it is getting boring.”

### Data collection procedure

This study was comprised of three different phases. In the first phase of the study, the participants were asked to complete the questionnaires on boredom to measure the level of this emotion before the intervention. In the second phase of the study, the researcher implemented 10-week intervention program in which approaches of autonomy enhancement were employed with the intention of reducing boredom. Each session of the program focused on one approach including *resource-based approaches, learner-based approaches, classroom-based approaches*, and *curriculum-based approaches* (Benson, 2011). Each approach was fully implemented via different tasks during the class in order to increase autonomy and diminish the negative states of boredom. *Resource-based approaches* provide learners with opportunities to direct their own learning and emphasize independent interaction with learning materials, *learner-based approaches* focus on learner development and changes within the learner, classroom*-based approaches* attend to the control learners have over the learning process including planning and evaluation, and finally, *curriculum-based approaches* emphasize learner control over the curriculum and learning material (Benson, 2011). Finally, in the third phase, after the intervention program was finished, learners were asked to complete the questionnaires on boredom again in order to make comparison and see whether any changes had been made in the level of this negative state.

As for the processes through which learners developed their autonomy and reduced their boredom as a result of taking part in each session of the intervention, they were required to reflect on their experiences after each session of the intervention. The questions included: *How did you feel during the class? Talk about your positive and negative emotions during the class, did you feel engaged in the tasks? What did you like about the activities and classes? Did you feel bored? If yes, please describe your feeling, etc.* The reflections were in form of written dairies which were sent to the researcher through Telegram in a weekly schedule.

### Autonomy-oriented intervention program

One approach to fostering autonomy is classroom-based approaches which may include different instructional activities such as peer teaching, project works, and peer assessment. The review of literature indicates that lack of cooperation among learners is a crucial factor in students’ boredom (Yazdanmehr et al., 2021; Zawodniak et al., 2021). Therefore, adopting classroom-based approaches such as pair work, group work, and project-based approaches would foster learner autonomy. In this study, classroom-based approaches are operationalized in terms of pair work and group work in which students need to do different activities. In online adobe connect classes, there is the possibility of breaking the class into groups and at the same time monitoring their activities. In the first session of the pair work, students were asked to divide the reading into two sections and each student summarized one section and explained to her/his partner. Then they were asked to do the comprehension questions in pairs. Finally, the answers to questions were checked in the class as a whole. In the second session of this approach, the class was divided into seven groups. First, some discussion questions were raised and then the students had to discuss them in their groups. While they were doing the task, the teacher observed the rooms to ensure their participation and passed her comments if necessary.

Moreover, the fact that students have no choice in English classes have been an important matter in making students feel bored (Pawlak et al., 2020a; Yazdanmehr et al., 2021; Zawodniak et al., 2021). Therefore, adopting curriculum-based approaches in which students can have a role in determining the content of the classes can make them feel autonomous and give them the right to choose their learning material (Benson, 2011). Curriculum-based approach has been conceptualized via adopting process-based syllabus through which learners play an important role in deciding the content and process of the class (Benson, 2011). In the present study, students are involved in developing process-based syllabus for two sessions based on their language needs and interest. That said, learners negotiated the content of classroom. In doing so, different poles were run in order to determine the general area which they preferred to work on. Because the coursebook taught at university is mainly reading-based, most of them selected grammar and listening skills to work on. Then, the general area of grammar was narrowed down to several parts including tenses, conditionals, modals, clauses, etc. As for two sessions of this approach, the instructor taught some grammatical points to the students via adopting task-based language teaching. Regarding the listening skill, the researcher assigned a task to work on their listening and also enhance learner-centered approaches. In doing so, students were divided to seven groups and each group had to choose a Ted Talk relevant to the content of the reading passage of the coursebook. In their groups, they had to watch the Talk, work on the new words, summarize it and then the leader of the group presented the main points to the class.

Resource-based approaches are important in fostering learners’ autonomy since using extracurricular activities and task could be a way to reduce boredom (Yazdanmehr et al., 2021). Hill and Hannafin (2001) defines resources as the “media, people, places, or ideas that have the potential to support learning” (p. 38) and the effectiveness of these resources is interconnected with their potentials to offer collaborative experiences and the sense of engagement with other people in learning process (Benson, 2011). During the intervention sessions, different resources were utilized. For instance, one session was allocated to watching a 15-mins video clip about the content of the unit (how civilizations end). The students were asked to watch the video clip and then answer some questions. First the questions were answered individually then they were asked to discuss the content of the videos in groups. Moreover, in order to draw on technological affordances in EAP classes, students had to do certain tasks and then share with the instructor on Telegram messaging application. For instance, they were required to share their ideas about one of the topics (Social robots, exploring space, etc.) and record their voices then share them via telegram. The other session was allocated to analyzing a piece of newspaper in the class. Each group could bring their own newspaper to the class so that they can analyze the structures and new words utilized in the newspaper.

Among the main causes of boredom, we can refer to teacher-centered classes in which students get bored as a result of long teacher talk, teacher control over classroom procedure, and teachers’ instructional practices (Derakhshan et al., 2021; Yazdanmehr et al., 2021; Zawodniak et al., 2021). Therefore, moving from teacher-centered to learner-centered approaches would help alleviate students’ boredom. Learner-centered approaches which are among autonomy enhancement techniques utilize a variety of activities to promote autonomy (Benson, 2011) and in turn reduce boredom. In this present study, learner-centered approaches are operationalized through adopting task-based teaching which are valid methods for promoting autonomy (Viera, 2017). The inclusion of tasks into the traditional syllabi would provide opportunities for communicate language use which in turn would enhance autonomy (Bygate, 2016). During each session of the intervention, different tasks such as watching video clips, analyzing newspapers, focusing on Ted Talks, discussing different topics were implemented. For each task, the three phases of pre-task, while task, and post-task activities were considered and implemented. The summary of the approaches utilized are provided in Table 1.

**TABLE 1:** Summary of autonomy-enhancement approaches.

Autonomy- enhancement approaches	Content and procedure
Classroom-based approaches	Implementing different pair work and group work activities for different sections of the class namely reading comprehension activities, discussion sections, etc.
Curriculum-based approaches	Developing process-based syllabus by giving the students the chance to determine the content of the based on their needs and interests. The listening skill and grammar were selected by the students to be included in the syllabus
Resource-based approaches	Drawing on various resources namely audio-visual medias for discussion in the class, technological affordances such as interaction through social medias, analyzing authentic texts, etc.
Learner-based approaches	Implementing Task-based teaching by inclusion of different tasks such as watching topic-based videos, analyzing authentic texts like newspapers, introducing and working on Ted Talks, etc.

### Data analysis

First, descriptive statistics were conducted to measure the rate of boredom before and after the intervention. The mean of each questionnaire was calculated before and after the intervention to be able to compare the scores and the possible changes in the level of boredom. Following that, paired sample *t*-test was run to see whether any significant differences are found between pre- and post-test scores of boredom as a result of taking part in the intervention.

Since we aimed to explore how intervention experiences had affected students’ boredom and how their autonomy was enhanced during the intervention sessions, we engaged in analyzing the reflective journals written by them based the guidelines of qualitative data analysis through thematic analysis (Braun and Clarke, 2006). They introduced six stages of inductive analysis including familiarizing yourself with your data, generating initial codes, searching for themes, reviewing themes, defining and naming themes and producing the report. Following these six steps, we initially collected the data inductively to discover the general themes of the journals and then it was coded deductively by drawing on the theoretical background of the study including the approaches to autonomy enhancement. As the second researcher was the instructor who implemented the intervention and also collected the data, she had already engaged in making sense of students’ experiences and feelings. However, the first researcher cross-checked the data and interpretations by the second researcher to confirm the analyses.

## Results

The result of this study is reported in two separate sections including quantitative and qualitative results.

### Quantitative results

The research question explored the differences between students’ boredom before and after the intervention. To achieve this purpose, the overall mean score of students’ pre-test on boredom is compared to the overall mean score of their post-test.

A paired *t*-test was used to compare the pre and post-tests results of the participants. The results indicates that there was a significant difference in the scores of pre-test (*M* = 68.4, SD = 8.1) and posttest (*M* = 65, SD = 6.8), *t*(2.72) = 0.008, *p* < 0.001 (two-tailed) (Table 2). Regarding the effect size, Cohen’s *d* is reported to be 1.14 indicating a large effect size.

**TABLE 2:** Paired sample *T*-test of pre- and post-test scores of participants.

Paired samples test									
		Paired differences							
		Mean	Std. deviation	Std. error mean	95% Confidence interval of the difference		*t*	d*f*	Sig. (2-tailed)
					Lower	Upper			
Pair 1	PreTest–PostTest	3.47059	11.76515	1.27611	.93290	6.00827	2.720	84	0.008

The descriptive and the paired samples *t*-test results revealed that the students’ boredom reduced as a result of adopting the autonomy-oriented program. These results showed that the application of this intervention program has significantly impacted the students’ boredom. Moreover, these results are strongly corroborated by the qualitative data obtained from reflective journals written by the participants. In the following section, the results of the qualitative data are reported.

### Qualitative results

This section reports the result of qualitative data gathered through reflective journals. The four dominant themes were classroom-based, curriculum-based, resource-based, and learner-based experiences. In the following sections, these experiences are introduced and the related sub-themes are provided with the participants’ excerpts.

#### Classroom-based experiences

The students’ journals revealed that they were satisfied with new approaches and they were mainly engaged in learning processes in online EAP classes. Regarding the classroom-based approaches, a number of subthemes including, collaboration and socialization and psychological affects were extracted.

##### Collaboration and socialization

As a result of attending online university classes, students could communicate and interact less than face-to-face classes. However, by adopting strategies such as pair work and group works or presentations, which helped them have communicative interactions with each other, they felt more active and involved:

**Participant 11:** We were more intimate in these groups, no stress, a friendly atmosphere in which we could learn.
**Participant 54*:*** We hadn’t met each other face to face, so first we didn’t know to how to communicate with each other, but little by little we knew how to move on and do the tasks.

They were also assigned different tasks which required their collaboration and cooperation. For instance, they needed to help each other with the correct pronunciation of the new words “*Breakout room made the class more productive, I guess. We certainly were more engaged. We were involved in reading the passage and interpreting it*” (Participant 45). “*We had more cooperation and interaction and helped each other through difficult tasks or pronunciation of different words*” (Participant 32).

##### Psychological affects

Taking part in group and pair work activities helped students manage their stress because they were working with their classmates and friends who were at the same social status as them. The students said that:

**Participant 10:** Sometimes we are shy to ask questions in front of everyone but in our groups, we could do it easier.
**Participant 72:** I’m not good at English but today’s class was more comprehensible for me, I also felt more confident, I thought they are my friends so it was easier.

Moreover, they could use sense of humor in the groups to make the activity more interesting. As a result of making the class funnier, they believed they would feel less bored: “*It was really fun; I had little stress; we could joke around with the classmates and enjoy the moments.*” (participant14), “*my classmates used their sense of humor in the class and this kept me interested and engaged in the activities*” (participant 31).

#### Curriculum-based experiences

The students were highly involved in classroom procedures through the curriculum-based approaches adopted by the instructor. A number of themes including feeling autonomous and novel university classes were extracted.

##### Feeling autonomous

In parallel with the quantitative results, some learners reported that having control over the content of the classroom or tasks protected them against feeling bored as the following extracts from the journals show:

**Participant 15:** I felt as if I am an independent person who can decide what to learn based on her own needs.
**Participant 82:** I had the power to choose whatever I want. I wasn’t forced to study s.th imposed by the university and this made me feel autonomous.
**Participant 19**: We had more autonomy to choose how we can manage out time, we could allocate more time to the sections which we thought was more difficult.

##### Novel university classes

The participants believed that it’s typical for the instructor of the course to choose the material and design the syllabus and the students need to follow the pre-determined materials in the class. However, the EAP classes have been totally different from other university classes which made it appalling for students: “*I felt like this was not a university class, so I willingly attended the classes each session*” (Participant 47*);* “*The class was interesting and exciting, it was different from normal university classes*” *(participant 64).* The fact that they have been considered as important individuals during the class time made them feel motivated: “*Thinking that the prof cares about the students*’ *choices and helps them make progress in their weaknesses is awesome*” (Participant 22)*; “It*’*s a great feeling to think you are important in the class procedure and the fact that the prof pays attention to our needs makes us motivated*” (Participant 77).

#### Resource-based experiences

The participants of this study considered resource-based approaches as real life activities and entertainment and engagement, which contributed to their involvement in online classroom procedure and prevented them from succumbing to boredom.

##### Real life activities

Drawing on different sources to be utilized in online classes made the students feel they were doing useful tasks which would contribute to the knowledge they need in real life: “*It was like real life activities that I will face in real life. I had to try to understand authentic texts or videos*” (Participant 59); “*I found the activities useful for my future because I was getting familiar with the real English used by native speakers”* (Participant 8). Moreover, they believed bringing new sources to the classroom creates challenge for them, which requires their hard work and attention during the classes: “*Listening sources were added, watching a movie was great, the struggle I was faced with made me try more”* (Participant 3); “*I had to understand the content of the newspaper, so I had to try more, look up the new words, ask more questions*” (Participant 80).

##### Entertainment and engagement

Utilizing different resources during the online classes made the students entertained and engagement because they believed the variety included in the materials to be worked on added sense of excitement and engagement to the classes as the following journal extracts show:

**Participant 55:** Novel and unexpected activities were included so it engaged me a lot, during the class I normally checked my cellphone but, in this class, I was focused on what was going on.
**Participant 46:** It wasn’t boring, I didn’t consider it as a class which bores me, I thought this was a group meeting in which I can learn new things!
**Participant 62:** Honestly, cool, I wish other classes had the same entertaining sessions.

#### Learner-based experiences

Adopting different tasks helped alleviate the boredom experienced in online EAP classes. The participants referred to some advantages including time passing quickly and strategy development.

##### Time passing quickly

The students believed that while they were doing different activities, the time passed quickly since they were engaged in the learning process through different activities which demanded their active participation in the classes as the following extracts reveal:

**Participant 84:** Time passes quickly in these classes because I’m engaged in doing the tasks assigned to me.
**Participant 70:** I keep checking the time in normal classes but time passes so slowly; however, when I first looked at my watch, I noticed half of class had passed because I was fully involved in the task.

##### Strategy development

Application of learner-based approaches lead to strategy development of students since they were in charge of controlling the classroom procedure. When faced with difficulties, they could draw on different strategies such as checking their dictionaries, using their L1 for better comprehension, etc. as the excerpts below indicate:

**Participant 29:** We used different dictionaries to check the words in our groups when none of us knew the meaning.
**Participant 33:** We could even use Persian equivalents, which was only possible in our groups not in the class as a whole.

## Discussion

The findings indicated positive effects of the autonomy-oriented intervention on learners’ boredom in EAP classes, which was caused through four different approaches including classroom-based, curriculum-based, resources-based, and learner-based approaches. The significant decline in learners’ boredom can be attributed to the tasks which were applied during each session of the intervention. In addition to the statistical results, the qualitative data gathered weekly contributed to our understanding of the learners’ experiences throughout the intervention. The qualitative data allowed a much more fine-grained understanding of how students were able to manage this negative emotion. The themes and subthemes highlight the prominent role of pedagogical interventions on mitigating negative emotions during online courses (Derakhshan et al., 2021).

Boredom of students in online classes could be explained in terms of teachers long and monotonous talks in the classes (Derakhshan et al., 2021; Pawlak et al., 2021). The results showed that providing students with opportunities through which they can experience peer teaching and active participation in the classes could contribute to reduction of the monotony of the class. This shred of evidence is supported by cooperative learning tasks through which teacher assigns different responsibilities to the learners and monitors them while conducting the tasks, which would lead to arousing positive emotions in learners (Mennim, 2017; Pawlak et al., 2021). In addition, due to the lack of interaction and cooperation in online classes, students succumb to boredom (Derakhshan et al., 2021; Yazdanmehr et al., 2021). However, using different approaches such as pair works and group works created resemblance to face-to-face classes in which students had great interactions with each other. As indicated in their journals, they had a sense of socialization to their community when engaged in pair work tasks. Moreover, adopting classroom-based approaches contributed to the psychological status of learners. Since they were interacting with their friends, they did not fear to make mistakes. In addition, they added a sense of humor to the tasks, which is in line with other studies (Derakhshan et al., 2021; Pawlak et al., 2021) which deem humor and fun as factors reducing boredom in online classes.

Equally important is students’ choice in the content and procedure of the classroom. According to Pekrun et al., 2010, p. 533, bored students have “lower perceived control” over their learning process. This can be explained with reference to control-value theory of achievement emotions (Pekrun, 2006; Pekrun et al., 2010) which highlights the fact that when lacking control over the learning process, students will feel bored. Therefore, if we provide them with freedom of choice, they perceive themselves in control and accordingly, feel less bored (Yazdanmehr et al., 2021). This is supported by Yazdanmehr et al.’s study in which the case participant attributed boredom to teacher control over the class which would cause disengagement in online courses. In addition, this is corroborated by findings of the study conducted by Pawlak et al. (2021) whose participants found content-based classes less boring in comparison to lecture-based presentations since they had more freedom to choose the content of the class. The participants of this study believed by choosing the content, they will be more autonomous which is in line with Benson’s (2011) approach to fostering autonomy namely curriculum-based approaches. Moreover, these approaches adopted during the online classes were less similar to typical university classes which seemed novel to the students and made them more engaged and less susceptible to boredom. As Pawlak et al., 2021, p. 19 remarks being “less reliant on university level English instruction” can made students more autonomous and alleviate their boredom in online classes.

Regarding the role of resource-based approaches in reduction of autonomy, learners were satisfied with the fact that they are using different resources with which they will be faced in out of class activities. This is in line with the findings of Pawlak et al. (2021) who found that relating tasks to real life activities will make the instruction more practical and reduce students’ boredom in online classes. However, when the tasks have nothing or little to do with real life activities, students feel bored (Zawodniak et al., 2021). Using authentic materials such as analyzing newspapers or Ted Talks also leads to reduction of boredom (Benson, 2011; Derakhshan et al., 2021). One important antecedent of boredom is reported to be disengagement of learners (Pawlak et al., 2020a). Therefore, provision of different resources which seems engaging and entertaining would mitigate boredom among university students (Pawlak et al., 2020a) since preventing disengagement is a direct strategy for overcoming boredom (Macklem, 2015). In the same vein, teachers in Pawlak et al.’s (2021) study utilized different strategies among which increasing students’ engagement was the most popular one to mitigate boredom. In addition, resource-based approaches provide opportunities for developing autonomy and control over the learning process through combination of self-access materials and class material to expand different skills in learners (Benson, 2011). Inclusion of various sources such as online resources can also be beneficial in mitigating boredom as well (Pawlak et al., 2021).

On the other side, the tasks utilized in the class play a pivotal role in alleviating students’ boredom. According to the participant’s journals, when the instructor adopted new tasks, they were engaged and this resonates well with the studies which regarded repetitive tasks as main indicator of students’ boredom (Derakhshan et al., 2021; Yazdanmehr et al., 2021). In the same vein, Zawodniak et al. (2021) also remarks that similarity in the tasks leads to feeling bored therefore, adopting novel approaches would provoke students’ engagement and reduce their boredom. Moreover, if instructors can adopt different pedagogical instructions and refrain from repetitious teaching methods, boredom would diminish in students in online courses (Derakhshan et al., 2021). Furthermore, tasks can offer communicative contexts in which students get involved in language learning and therefore foster their autonomy (Bygate, 2016; Viera, 2017). Task-based language teaching promotes learner-centeredness which requires taking one step away from traditional teaching methods and moving forward to learner empowerment and autonomy enhancement (Viera, 2017). As implied by qualitative analysis, learners were able to develops strategies for learning language which is a way of developing autonomy in learners (Benson, 2011), which leads to reduction of boredom (Mercer and Dörnyei, 2020). As Harmer, 2007, p. 394 remarks, learner-centered approaches help learners to be “the doers rather than the recipients of learning action,” which results in higher positive emotions and lower negative emotions. Moreover, learners posited that time passed by quickly during the learner-based approaches while one major factor for boredom is considered as the perceptions of time slowing down (Sharp et al., 2016).

## Conclusion

This study was designed to investigate the effect of an autonomy-oriented intervention program on reducing boredom among EAP students. The findings of qualitative and quantitative measures revealed significant changes in the boredom of learners before and after our intervention. The study, therefore, has brought to the fore the need for online English courses to be delivered in a more engaging manner which utilizes different approaches to foster the autonomy of learners so that they can take charge of their learning and as a result overcome their boredom.

The present study offers pedagogical approaches helpful in reducing boredom among EAP learners at university levels. Introducing variety and avoiding repetitive tasks in the classroom provides ample opportunities for engaging students since task repetition over a long period of time leads to less engagement and eventually boredom of students. Moreover, teachers should refrain from acting authoritative in the classes and do their best to encourage involvement during different activities.

Due to COVID-19 pandemic and other unforeseen disastrous pandemics, there must be attempts to enhance the quality of online classes. The inadequacies of online courses should be dealt with through strategic instructions which engage students in various types of tasks. Moreover, by keeping balance between adopting different approaches such as whole class, pair work, group work mode would lead to reduced boredom (Zawodniak et al., 2021).

## Limitations and future avenues

Findings of the present study along with the pedagogical instructions employed during the intervention could aid learners and teachers in mitigating their negative emotions during online courses. However, the findings should be interpreted with circumspection since the individual factors and their roles on students feeling has not be considered. Future studies should take a further step to explore the effect of individual differences on how students feel bored and how they would react to different pedagogical interventions. Moreover, the enhancement of autonomy requires instructors who are familiar with the autonomy enhancement approaches since this process is “a more active process of guidance and encouragement to help learners extend and systematize the capacities that they already possess” (Benson, 2011, p. 91). Another significant limitation with which this study was faced is related to the lack of control group since having two groups of participants would yield more accurate results. Therefore, caution must be taken in the interpretation of the results and future studies might include a control group for obtaining more generalizable and accurate results.

## Data availability statement

The original contributions presented in this study are included in the article/supplementary material, further inquiries can be directed to the corresponding author.

## Author contributions

All authors listed have made a substantial, direct, and intellectual contribution to the work, and approved it for publication.
